# Paradox of schizophrenia genetics: is a paradigm shift occurring?

**DOI:** 10.1186/1744-9081-8-28

**Published:** 2012-05-31

**Authors:** Nagafumi Doi, Yoko Hoshi, Masanari Itokawa, Takeo Yoshikawa, Tomoe Ichikawa, Makoto Arai, Chie Usui, Hirokazu Tachikawa

**Affiliations:** 1 Ibaraki Prefectural Medical Center of Psychiatry, 654Asahi-machi, Kasama-shi, Ibaraki, 309-1717, Japan; 2 Integrated Neuroscience Research Project, Tokyo Metropolitan Institute of Medical Science, 2-1-6 Kamikitazawa, Setagaya, Tokyo, 156-8506, Japan; 3 Project for Schizophrenia and Affective Disorders Research, Tokyo Metropolitan Institute of Medical Science, 2-1-6 Kamikitazawa, Setagaya, Tokyo, 156-8506, Japan; 4 Laboratory for Molecular Psychiatry, RIKEN Brain Science Institute, 2-1Hirosawa, Wako-shi, Saitama, 351-0198, Japan; 5 Department of Psychiatry, Juntendo University Nerima Hospital, 3-1-10 Takanodai, Nerima-ku, Tokyo, 177-8521, Japan; 6 Department of Psychiatry, Graduate School of Comprehensive Human Science, Tsukuba University, Tsukuba-shi, Ibaraki, Japan

**Keywords:** Mutation-selection balance, Heterozygote advantage, Sex difference, MtDNA, Gene-gene interaction, Gene-environment interaction, Protective gene, Mitochondrial dysfunction, Oxidative stress, Genomic instability

## Abstract

**Background:**

Genetic research of schizophrenia (SCZ) based on the nuclear genome model (NGM) has been one of the most active areas in psychiatry for the past two decades. Although this effort is ongoing, the current situation of the molecular genetics of SCZ seems disappointing or rather perplexing. Furthermore, a prominent discrepancy between persistence of the disease at a relatively high prevalence and a low reproductive fitness of patients creates a paradox. Heterozygote advantage works to sustain the frequency of a putative susceptibility gene in the mitochondrial genome model (MGM) but not in the NGM.

**Methods:**

We deduced a criterion that every nuclear susceptibility gene for SCZ should fulfill for the persistence of the disease under general assumptions of the multifactorial threshold model. SCZ-associated variants listed in the top 45 in the SZGene Database (the version of the 23^rd^ December, 2011) were selected, and the distribution of the genes that could meet or do not meet the criterion was surveyed.

**Results:**

19 SCZ-associated variants that do not meet the criterion are located outside the regions where the SCZ-associated variants that could meet the criterion are located. Since a SCZ-associated variant that does not meet the criterion cannot be a susceptibility gene, but instead must be a protective gene, it should be linked to a susceptibility gene in the NGM, which is contrary to these results. On the other hand, every protective gene on any chromosome can be associated with SCZ in the MGM. Based on the MGM we propose a new hypothesis that assumes brain-specific antioxidant defenses in which trans-synaptic activations of dopamine- and *N*-methyl-d-aspartate-receptors are involved. Most of the ten predictions of this hypothesis seem to accord with the major epidemiological facts and the results of association studies to date.

**Conclusion:**

The central paradox of SCZ genetics and the results of association studies to date argue against the NGM, and in its place the MGM is emerging as a viable option to account for genomic and pathophysiological research findings involving SCZ.

## Background

Schizophrenia (SCZ) is a common and highly heritable form of psychosis associated with a remarkable biological disadvantage [1]. Accordingly, the central paradox of SCZ genetics is how susceptibility genes are preserved in the human gene-pool against a strong negative selection pressure [2-5]. Although recent large-sample epidemiological studies [6-9] have consistently shown that the reproductive fitness of the unaffected *female* siblings of patients with SCZ is slightly increased (1.02-1.08), it is not large enough to compensate for the gene loss due to the decreased reproductive fitness of patients (0.2-0.3 in males and 0.4-0.5 in females) and their unaffected male siblings (0.9-1.0) in the nuclear genome model (NGM). Furthermore, the latest meta-analysis shows that parents of patients with SCZ have fertility similar to the general population [10]. Therefore, heterozygote advantage does not seem to work in the NGM.

However, it works in the mitochondrial genome model (MGM) because mtDNA is transmitted to the next generation only through females. Indeed, we can see that this slightly elevated fitness of the unaffected female siblings, coupled with the less pronounced decreased fitness of female patients, is sufficient to compensate for the gene loss in the MGM; when we calculate –Δ, the cross-generational reduction of the frequency of females carrying putative pathogenic mtDNA in the general population, using data from the largest-sampled cohort study to date [8], we have $-\Delta <5.06\times 10^{-3}$[11]. This figure implies that the gene loss can be balanced by *de novo* mutation in the mtDNA which occurs at a rate of $8.8\times 10^{-4}∼1.3\times 10^{-2}$ per locus per generation (4.3 × 10^-3^ on average) [12].

Thus, in our previous paper [11], we carefully re-examined the necessary conditions for putative nuclear susceptibility genes for SCZ and deduced a criterion (the persistence criterion, or ‘P-criterion’) for nuclear susceptibility genes for SCZ based on the general assumptions of the multifactorial threshold model. Because SCZ must be sustained by mutation-selection balance without heterozygote advantage in the NGM and the mutation rates in the nuclear genome are much lower than in the mitochondrial genome [12-14], the criterion is strong. It demands $0<d<\nu \equiv \frac{(1-sp)\mu }{(1-p)sp}$ (Appendix A), while the principle of association study demands *d>*0. Here *dμp* and *s* denote the case–control difference of allele frequencies, the mutation rate at a putative risk locus, and the prevalence and the selection coefficient of the disease respectively. According to epidemiological data collected by Haukka et al. [8]$\left(p=1.29\times 10^{-2}\;\text{and}\;s=6.54\times 10^{-1}\right)$*ν* is very small for SCZ. Given the average mutation rate in the autosomes and the X chromosome $\left(\mu =1.48\times 10^{-5}\right)$, we have $\nu =1.76\times 10^{-3}$, and given the highest mutation rate $\left(\mu =1.48\times 10^{-4}\right)$, we have $\nu =1.76\times 10^{-2}$(Appendix B).

Using this criterion, we can calculate a lower boundary for the sample size required in an association study of a given power (Appendix C). Thus, we can see that more than 29,000 case–control pairs are required in a genome-wide association study (GWAS) with a power of 0.8 to detect a susceptibility variant of the highest mutation rate and a population frequency between 0.005 and 0.995, and that more than 2.9 million case–control pairs are required in a GWAS with a power of 0.8 to detect a susceptibility variant of the average mutation rate and a population frequency between 0.0005 and 0.9995.

In the past two decades, mitochondrial dysfunction (MD) in SCZ has been suggested by several independent lines of evidence [15-18], that include mitochondrial hypoplasia, elevated oxidative stress (OS), disturbed oxidative phosphorylation (OXPHOS), and altered mitochondrial-related gene expression in several cell lines. It is still unknown whether MD in the brains of patients with SCZ is a primary pathogenic change or a secondarily induced process, and the aforementioned inconclusive findings create more questions than answers about the origin of SCZ. We contend, however, that the MGM may very well have the answers.

In the present study, we apply the P-criterion to the results of association studies to date and survey the distribution of SCZ-associated genes that could meet or do not meet the P-criterion. We discuss why those SCZ-associated genes that do not meet the criterion must be protective genes (PGs) for SCZ, and suggest that the MGM can replace the NGM as a basis for genomic research of SCZ. Based on the MGM we propose a new hypothesis that assumes brain-specific antioxidant defenses in which neurotransmissions are involved, and present ten predictions of the hypothesis.

## Methods

SCZ-associated variants listed in the top 45 in the SZGene Database [19] (the version of the 23^rd^ December, 2011) were selected. Based on the genotype distributions in the meta-analyses, allele frequencies and the case–control differences were calculated.

## Results and discussion

### Distribution of SCZ-associated variants that could meet the P-criterion

Association studies to date have detected many SCZ-associated genes on various chromosomes [19,20]. However, most of these do not meet the criterion; only 21 variants on18 loci in the top 45 genes in the SZGene Database could meet the criterion under the assumption that the mutation rates at those loci are near the upper limit on the autosomes and the X chromosome (Tables 1 and 2). In other words, association studies have detected only *a few* susceptibility genes in the NGM, if any, that produce *small effects* (in most cases OR < 1.4). Association studies to date (sample size <50,000 case–control pairs) may lack the power to detect pathogenic variants of less than the highest mutation rates. The question remains: how many samples would be required to detect them?

**Table 1:** **SCZ-associated variants on chromosome 1 that could meet or do not meet the criterion in the Top 45 genes at the SZGene Database**[19]**(Updated Dec 23, 2011)**

**Genes and SNPs**	**Location**	**Allele (min/maj)**	***m***_***A***_	***m***_***U***_	***OR***	***d***
*MHTFR*	1p36.22					
rs1801133		T*/C	0.3532	0.3211	1.15	0.032
*GRIK3*	1p34.3					
rs6691840		G*/T	0.2600	0.2226	1.25	0.037
*PDE4B*	1p31.3					
rs910694		C/T*	0.5780	0.5477	1.30	0.030
*GSTM1*	1p13.3					
GSTM1*0		ins-allele/ del-allele*	0.7546	0.7140	1.35	0.041
***RGS4***	**1q23.3**					
**rs2661319**		A/G*	**0.4920**	**0.4744**	**1.08**	**0.0176**
*IL10*	1q32.1					
rs1800896		G*/A	0.3056	0.2657	1.42	0.040
***PLXNA2***	**1q32.2**	A/G				
rs841865		A/G*	0.8434	0.8001	1.32	0.043
**rs1327175**		G/C*	**0.92840**	**0.91243**	**1.32**	**0.016**
***DISC1***	**1q42.3**					
**rs3737597**		A*/G	**0.03069**	**0.01735**	**1.80**	**0.013**
**rs999710**		A*/G	**0.3989**	**0.3819**	**1.07**	**0.0170**

Four variants at the three loci (*RGS4*, *PLXNA2*, *DISC1*) on chromosome 1 could meet the criterion under the assumption that the mutation rates at those loci are near the upper limit in the autosomes and the X chromosome. All of them are located on 1q, while four protective genes (*MHTFR*, *GRIK3*, *PDE4B*, *GSTM1*) are on 1p.

*alleles associated with SCZ.

**Table 2:** SCZ-associated variants on chromosome 2-22 and the chromosome X that could meet or do not meet the criterion in the Top 45 genes at the SZGene Database (Updated Dec 23, 2011)

**Genes and SNPs**	**Location**	**Allele (min/maj)**	***m***_***A***_	***m***_***U***_	***OR***	***d***
*IL1B*	2q13					
rs16944		T/C*	0.6289 (N=1,718)	0.6066 (N=2,157)	1.11	0.022
*ZNF804A*	2q32.1					
rs1344706		G/T*	0.6357 (N=6,487)	0.6032 (N=11,478)	1.14	0.033
***CCKAR***	**4p15.2**					
**rs1800857**		C*/T	0.1666 (N=105)	0.1559 (N=93)	**1.32**	**0.008**
*GABARB2*	5q34					
rs1816072		C/T*	0.6404 (N=1,129)	0.5935 (N=995)	1.22	0.047
rs194072		C/T*	0.8685 (N=1,137)	0.8466 (N=991)	1.20	0.022
rs6556547		T/G*	0.9444 (N=774)	0.9226 (N=620)	1.43	0.022
***DTNBP1***	**6p23**					
**rs3213207**		G/A*	**0.8891 (N=8,377)**	**0.8758 (N=8,886)**	**1.11**	**0.013**
**rs1474605**		G/A*	**0.8000 (N=3,710)**	**0.7858 (N=3,588)**	**1.09**	**0.014**
***HIST1H2BJ***	**6p22.1**					
**rs6913660**		A/C*	**0.8927 (N=10,065)**	**0.8801 (N=34,098)**	**1.15**	**0.013**
***PRSS16***	**6p22.1**					
rs6932590		C/T*	0.7943 (N=7,177)	0.8058 (N=28,270)	1.16	-0.012
**rs13219354**		C/T*	**0.9088 (N=6,478)**	**0.9020 (N=27,224)**	**1.20**	**0.007**
***PGBD1***	**6p22.1**					
**rs13211507**		C/T*	**0.9518 (N=9,774)**	**0.9387 (N= 33,694)**	**1.23**	**0.013**
*RPP21*	6p21.33					
rs3130375		A/C*	0.9018 (N=2,799)	0.8634 (N=3,082)	1.41	0.038
***NOTCH4***	**6p21.32**					
**rs3131296**		A/G*	**0.8912 (N=7,156)**	**0.8892 (N=28,312)**	**1.20**	**0.002**
rs2071287		A/G*	0.6858 (N=2,511)	0.6463 (N=2,556)	1.19	0.039
***MDGA1***	**6p21.2**					
rs11759115		C/T*	0.8639 (N=1,874)	0.8379 (N=2,582)	1.23	0.026
**rs12191311**		T*/C	**0.3619 (N=1,879)**	**0.3555 (N=2,605)**	**1.12**	**0.006**
rs7769372		T*/C	0.2328 (N=1,915)	0.1885 (N=2,576)	1.18	0.044
***AH1***	**6q23.3**					
rs2064430		T*/C	0.5672 (N=2,796)	0.5296 (N=13,494)	1.13	0.038
**rs11154801**		A/C*	**0.6633 (N=3,298)**	**0.6576 (N=14,805)**	**1.09**	**0.006**
***C6orf217***	**6q23.3**					
rs10223338		T/C*	0.7158 (N=2,797)	0.6975 (N=13,522)	1.12	0.018
rs1475069		C*/A	0.7205 (N=3,276)	0.6976 (N=14,639)	1.14	0.023
**rs9321521**		A/G*	**0.6755 (N=2,784)**	**0.6642 (N=13,311)**	**1.12**	**0.011**
*RELN*	7q22.2					
rs7341475		A/G*	0.8327 (N=3,315)	0.8110 (N=8,042)	1.14	0.022
rs262355		A*/T	0.3852 (N=1,128)	0.3563 (N=1,848)	1.14	0.029
***PPP3CC***	**8p21.3**					
**rs2461491**		A*/G	**0.4469 (N=6,287)**	**0.4353 (N=6,200)**	**1.07**	**0.015**
**rs10108011**		G*/A	**0.3657 (N=4,304)**	**0.3488 (N=4,465)**	**1.09**	**0.0170**
*SLC18A1*	8p21.3					
rs2270641		C*/A	0.3182 (N=759)	0.2802 (N=885)	1.63	0.038
*NRG1*	8p12					
rs10503929		C/T*	0.8342 (N=3,256)	0.8118 (N=4,181)	1.14	0.022
GWA_10q26.13	10q26.11					
rs17101921		A*/G	0.06667 (N=7,447)	0.04318 (N=13,039)	1.28	0.023
*DRD4*	11p15.5					
rs4646984		S/L*	0.7750 (N=1,558)	0.7292 (N=1,499)	1.27	0.046
rs1800955		C*/T	0.4235 (N=2,450)	0.4018 (N=2,506)	1.12	0.022
*TPH1*	11p15.1					
rs1800532		A*/C	0.4752 (N=2,416)	0.4299 (N=3,623)	1.16	0.045
rs1799913		A*/C	0.4546 (N=1,323)	0.4171 (N=2,201)	1.14	0.038
GWA_11p14.1	11p14.1					
rs1602565		C*/T	0.1346 (N=5,475)	0.1152 (N=10,845)	1.19	0.019
***DRD*2**	**11q23.1**					
rs6277		C*/T	0.4968 (N=3,156)	0.4527 (N=3,960)	1.40	0.044
rs6275		T*/C	0.3106 (N=2,436)	0.2927 (N=2,918)	1.15	0.018
**rs1801028**		G*/C	**0.0245 (N=4,304)**	**0.0207 (N=5,920)**	**1.33**	**0.004**
*NRGN*	11q24.2					
rs12807809		C/T*	0.8403 (N=7,213)	0.8190 (N=28,490)	1.15	0.021
*OPCML*	11q25					
rs3016384		T/C*	0.5374 (N=5,491)	0.5141 (N=10,900)	1.10	0.023
*GRIN2B*	12p13.1					
rs1019385		T/G*	0.5604 (N=687)	0.4885 (N=650)	1.33	0.072
*DAO*	12q24.11					
rs4623951		C/T*	0.7038 (N=1,509)	0.6788 (N=1,521)	1.14	0.025
*HTR2A*	13p14.13					
rs6311		A*/G	0.4441 (N=2,594)	0.4115 (N=2,869)	1.21	0.033
*DAOA*	13q33.3					
rs778293		G*/A	0.3149 (N=2,899)	0.2770 (N=3,218)	1.18	0.038
rs3916971		T/C*	0.5622 (N=844)	0.5211 (N=922)	1.19	0.041
***AKT1***	**14q32.33**					
**rs3803300**		A*/G	**0.1068 (N=1,301)**	**0.1039 (N=1,424)**	**1.36**	**0.003**
GWA_16p13.12	16p13.12					
rs7192086		T*/A	0.2701 (N=7,179)	0.2456 (N=12,623)	1.12	0.025
***RPGRIP1L***	**16q12.2**					
**rs9922369**		A*/G	**0.0403 (N=6,494)**	**0.0336 (N=11,449)**	**1.32**	**0.007**
*HP*	16q22.3					
Hp_1/2		1/2*	0.6208 (N=1,300)	0.5911 (N=1,976)	1.14	0.030
*SRR*	17p13.3					
rs408067		G*/A	0.3166 (N=1,145)	0.2779 (N=1,146)	1.22	0.039
***TCF4***	**18q21.2**					
**rs9960767**		C*/A	**0.0514 (N=9,755)**	**0.0467 (N=33,648)**	**1.23**	**0.005**
*APOE*	19q13.32					
e4-allele		E2/3/4*	0.1391 (N=1,596)	0.1139 (N=3,038)	1.16	0.025
***COMT***	**22q11.21**					
**rs737865**		C/T*	**0.6916 (N=7,397)**	**0.6803 (N=10,411)**	**1.06**	**0.011**
rs4818		G*/C	0.3503 (N=177)	0.3081 (N=99)	1.05	0.042

On chromosomes 2-22 and the chromosome X, 17 variants at 15 loci (*CCKAR*, *DTNBP1*, *HIST1H2BJ*, *PRSS16*, *PGBD1*, *NOTCH4*, *MDGA1*, *AH1*, *C6orf217*, *PPP3CC*, *DRD*2, *AKT1*, *RPGRIP1L*, *TCF4*, *COMT*) could meet the criterion under the assumption that the mutation rates at those loci are near the upper limit in the autosomes and the X chromosome. 15 protective genes (*IL1B*, *ZNF804A*, *GABARB2*, *RELN*, GWA_10q26.13, *DRD4*, *TPH1*, GWA_11p14.1, *GRIN2B*, *DAO*, *HTR2A*, *DAOA*, GWA_16p13.12, *SRR*, *APOE*) are located on 2q, 5q, 7q, 10q, 11p, 12p, 12q, 13p, 13q, 16p, 17p, and 19q, where no SCZ-associated variants that could meet the criterion are located

*alleles associated with SCZ.

The P-criterion implies that an enormous sample size is required to identify a pathogenic variant of the average mutation rate. Indeed, more than 2.9 million case–control pairs should be recruited to a GWAS to identify a susceptibility variant of a population frequency between 0.0005 and 0.9995 with a power of 0.8 (Appendix C). If the mutation rate at a putative risk locus is relatively low $\left(\mu =1.48\times 10^{-6}\right)$, more than 290 million case–control pairs are required in a GWAS to detect a risk variant of a population frequency between 0.000005 and 0.999995 with a power of 0.8 (Appendix C). Since the total human population today is ~ 7 billion [21] and the overall prevalence of SCZ is ~ 0.7% [22], the total number of patients with SCZ in the world today is ~ 49 million. Therefore it would take more than several hundred years to gather the required number of samples even if all of the affected individuals in the world were to be recruited. The journey to search for susceptibility genes for SCZ in the NGM seems far more difficult and would take longer than previously thought.

### Distribution of SCZ-associated variants that do not meet the criterion

Now, let us consider the nature of those SCZ-associated genes that do not meet the criterion. The inequality *d*≥ *v *implies *s*_*M*_ *< s*, where *s*_*M*_ and *s* denote the selection coefficient in the affected subpopulation with an allele M and in the affected population as a whole respectively **(**Appendix A); otherwise, the frequency of M in the affected population must have been decreased to the same level in the unaffected population. Therefore, such genes, if sustained by mutation-selection balance, cannot be susceptibility genes but instead are PGs that decrease the risk and the severity of the disease.

Since a PG in the NGM cannot be associated with the disease unless it is linked to a susceptibility gene, PGs in the NGM should be located in the vicinity of susceptibility genes, something that is contrary to the results of association studies to date. For example, on chromosome 1, all of the SCZ-associated genes that could meet the criterion (*RGS4*, *PLXNA2*, *DISC1*) are located on 1q, while 4 PGs (*MHTFR*, *GRIK3*, *PDE4B*, *GSTM1*) are on 1p (Table 1). 15 PGs are located on 2q, 5q, 7q, 10q, 11p, 12p, 12q, 13p, 13q, 16p, 17p, and 19q, where no SCZ-associated variants that could meet the criterion are located (Table 2). Therefore, the results of association studies to date do not support the NGM.

The only plausible interpretation that accords with the NGM may be that many nuclear susceptibility genes of less than the highest mutation rates have not been detected by association studies to date due to lack of power. If we assume this case, however, an enormous sample size (more than 2.9 ~ 290 million case–control pairs) would be required to identify them. Although the possibility of the NGM cannot be entirely denied, the proof of the NGM may be extremely difficult because it requires such an enormous sample size.

### The MGM is compatible with the results of association studies to date

A case can be made for the MGM however, because *every* PG on *any* chromosome can be associated with SCZ in the MGM. This is because mtDNA is transmitted only via females and there is no link between the nuclear genome and the mitochondrial genome; such that every nuclear genome that interacts with a pathogenic mitochondrial genome to alter the risk and the severity of the disease is subject to natural selections in the predisposed maternal lineage that succeeds the same pathogenic mitochondrial genome. Therefore, in the MGM every PG on any chromosome is subject to positive selection in the predisposed maternal lineage, and is thereby associated with SCZ.

Thus, the MGM is compatible with the results of association studies to date.

It should be noted that in the MGM the frequency of every facilitating gene (FG; a gene that increases the risk and the severity of SCZ in the presence of a pathogenic mitochondrial genome) on any chromosome may diminish in the predisposed matrilineal pedigrees by negative selection; thereby, negatively associating with SCZ.

### mtDNA hypothesis for SCZ as an alternative genetic model

Based on the MGM, we propose a new genetic hypothesis for SCZ. It consists of two parts: H1 and H2.

**H1**: Pathogenic genes for SCZ are located in the mitochondrial genome and embryos from fertilized oocytes with mutation loads over a certain threshold can develop the disease. The threshold is determined by the interactions with the environmental and nuclear genetic factors.

**H2**: Disturbed OXPHOS and OS play central roles in the pathophysiology of SCZ, causing a variety of changes. Those include adaptive responses of the brain that are relevant to the exacerbation of the illness.

### Pathophysiological aspect of SCZ and the MGM

Mitochondria are involved in a variety of major cellular events such as OXPHOS, free radical production and Ca^2+^ buffering, and play an active role in apoptosis. They possess two classes of antioxidant defenses (non-enzymatic or enzymatic) [23], and structurally and functionally intact mitochondria serve as a *net sink* rather than a *net source* of reactive oxygen species (ROS). ROS-defenses are severely undermined in structurally compromised mitochondria [23], and MD, presumably through imbalance of ROS production and removal [23], raises ROS emission [24,25], causing a strong intracellular OS.

Mitochondrion has dual genetic bases (ncDNA and mtDNA), and there are 37 loci in the mtDNA (22 tRNAs, 2 rRNAs, and all of the 13 OXPHOS subunits: ND1, ND2, ND3, ND4, ND4L, ND5, ND6, CO1, CO2, CO3, CYB, ATPase6, and ATPase8). Therefore, abnormal mtDNA may cause disturbed OXPHOS and enhanced OS.

In the past decade, MD in SCZ has been suggested by several independent lines of evidence (for review, see [15-18]); those include increased OS, mitochondrial hypoplasia, disturbed OXPHOS, and altered mitochondrial-related gene expression in several cell lines.

The pioneering works in this field are noteworthy. As early as 1950, Hayashi [26], in a longitudinal study on glucose metabolites in blood sampled from the superior bulb of the internal jugular vein of patients with SCZ, observed a lower degree of carbonic dioxide production in the brain and a higher level of lactate and glutathione, the brain’s dominant free radical scavenger, in patients in an acute exacerbation of the illness. Utena and Ezoe (1951) [27] reported decreased glucose consumption *in vitro* in cortical brain tissues sampled from patients with SCZ who underwent prefrontal leukotomy. Takahashi (1953) [28] confirmed this finding and recommended further investigations on OXPHOS in the brain tissue of schizophrenics. In line with those findings was the report by Stabenau et al. (1969) [29], who observed in a biochemical study of discordant monozygotic twin pairs that lactate production and the lactate-pyruvate ratios were higher in the affected twins than in the unaffected co-twins. More recently, Prabakaran et al. (2004) [30], in a large-scale functional genomics study, suggested a state of intermittent or chronic hypoxic stress and MD in the brains of patients with SCZ.

There has been consistent evidence for higher incidence of underdevelopment of muscular system in patients with SCZ [31]. Many authors have confirmed the Kretchmer’s observation (1925) of a greater incidence of *asthenic* or *leptosomatic* constitution in schizophrenic or pre-schizophrenic individuals, which is characterized by narrowness of muscles, small shoulders, flat chest, and little muscular development [31]. These early observations have been supported by later studies of muscle biopsy and/or electromyography, which showed abnormalities of both muscle function (more hypotonus) and muscle morphology (atrophic muscle fibers scattered or in groups, regardless of the patients’ motor activities) in SCZ [32-36].

There also have been many case reports that describe patients with mitochondrial disease and neuropsychiatric disorders including SCZ (for review, see [37,38]). Most of the patients have psychotic symptoms as well as fatigue and muscle weakness that preceded the diagnosis of mitochondrial disease. Furthermore, it has been shown that mtDNA, in interaction with ncDNA, modifies learning, exploration, and sensory development as well as the brain morphology in mice [39].

Those findings indicate that abnormal mtDNA could underlie the etiology of SCZ.

The DA hypothesis and NMDA-R hypofunction theory as well as the recent finding of enhanced carbonyl stress in SCZ may be incorporated into the MGM as will be discussed in the following sections.

#### Contradictory aspects of dopamine-SCZ connection and the MGM

Substantial evidence has been accumulated that supports aberration in the dopamine (DA) system [40,41] and its interactions with other neurotransmitters [42] in SCZ. This concept (DA hypothesis of SCZ) mainly stems from indirect observations that there is a strong relationship between clinical potency of neuroleptics and their DAD2 receptor affinities *in vitro* and that DA agonists can induce psychotic symptoms with marked resemblance to SCZ. More recently, Laruelle et al. [43] reported that DA transmission was increased in patients experiencing an acute exacerbation of the illness, but not in patients in a state of remission.

Those findings strongly suggest that increased DA transmission provides a neuronal basis for positive or productive symptoms in SCZ. Since negative symptoms and cognitive impairments that can precede initial psychotic episode are difficult to explain by the increased DA transmission, a plausible interpretation may be that the increased DA transmission in the brain is a secondary process associated with SCZ. However, since excessive DA release seems to cause MD [16] and apoptosis [44], it could produce additional deterioration of the brain function and morphology after psychotic episodes.

On the other hand, Friedhoff and colleagues [45-47] reported some adaptive aspects of the DA system in SCZ, and proposed that the DA system may be a biological stress buffer. This concept is based on a consistent observation that patients responding favourably to neuroleptic treatment showed higher levels of plasma homovanillic acid (HVA), a metabolite of DA, in the pretreatment and/or the early treatment period, resulting in a marked decrease of plasma HVA level in a later steady state [46,48-50]. This observation suggests that increased DA turn over in the pre-treatment or early treatment period may be relevant to a certain adaptive process in patients with SCZ. In line with those findings is the discovery of Cys311 of DRD2 polymorphism [51], which is listed in the top 45 in the SZGene. This firstly discovered SCZ-associated variant supresses the internalization of DRD2 and strengthens the DA transmission [52] while the cases carrying this variant show less negative symptom and less cognitive impairment [51].

Then, how can we reconcile those two contradictory aspects of the DA-SCZ connection?

At low doses catecholamines, such as DA, noradrenaline (NA) and adrenaline (A), are thought to protect nerve cells by virtue of their antioxidant activities; whereas, at high doses they are thought to be neurotoxic, acting as pro-oxidants [44]. The protective effect of DA, but not of NA and A, has been shown to be through the activation of DA D4 receptors [53]. It is also shown that DA D2 and/or D3 receptor activation protects neurons [54] and/or oligodendrocytes [55] against OS. Thus it is suggested that DA, through the activation of D2, D3 and/or D4 receptors, may protect cells from gradual changes in low levels of OS that occur in mild pathological insults and aging [53-55]. Therefore, it seems not implausible to suppose that increased DA transmission in the brain could occur as an adaptive process against OS, especially in the individuals with MD and resulting vulnerability to OS.

Indeed, it has been shown that *in vitro* and *in vivo* treatment with the mitochondrial complex I inhibitor rotenone enhances the sensitivity of striatal DA release to OS induced by H_2_O_2_[56]. Thus, if MD and resultant vulnerability to OS underlie the pathophysiology of SCZ, positive symptoms of the disease might be explained as ‘the price that the brain pays for its homeostasis at OS’.

#### NMDA-R hypofunction theory of SCZ and the MGM

*N*-methyl-d-aspartate-receptor (NMDA-R) antagonists, such as phencyclidine and ketamine, produce SCZ-like symptoms including negative symptoms and cognitive impairments in healthy individuals [57-59], and exacerbate both positive and negative symptoms in patients with chronic SCZ [60-62]. In addition, NMDA-R antagonists induce disinhibition of neurotransmitter systems through specific effects on inhibitory circuits [63]. These indirect observations have led to the NMDA-R hypofunction theory of SCZ [42,64], which proposes that NMDA-R hypofunction provides a neuronal basis for the negative symptoms and cognitive impairments associated with SCZ and induce aberrations in the monoaminergic systems that could result in positive symptoms. Postmortem studies have revealed alterations in pre- and post-synaptic markers for glutamatergic neurons in several brain regions in patients with SCZ [64]. However, no conclusive evidence of NMDA-R hypofunction in SCZ has been obtained.

It has been shown that a rapid increase in ROS occurs *in vitro*[65] and *in vivo*[66] after exposure to NMDA-R antagonists. Exposure to ketamine suppresses the expression of GABAergic interneurons by inducing a persistent increase in brain superoxide through activation of NADPH-oxidase in the prefrontal cortex [63]; thereby, causing increased excitatory neurotransmission. Thus, the evidence for NMDA-R hypofunction theory may also support the hypothesis that OS plays a central role in the pathophysiology of the symptoms associated with SCZ.

**Figure 1:**
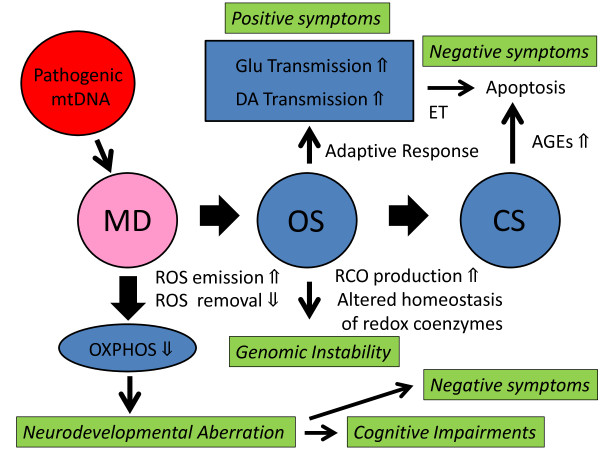
**Proposed pathophysiology of schizophrenia.** Mitochondrial dysfunction (MD), through imbalance of ROS production and removal, raises ROS emission, causing a strong intracellular OS. Disturbed OXPHOS and enhanced OS in predisposed individuals may cause various pathogenic alterations such as genomic instability, aberrations in neuromuscular development, brain dysfunction, and apoptosis. On the other hand, enhanced OS and secondarily induced alteration of redox coenzyme homeostasis may cause enhanced CS. It is assumed that the brain has the third class of antioxidant defenses in which neurotransmissions are involved, and that trans-synaptic activation of NMDA-R as well as DA-R may occur, through unknown pre-synaptic redox regulation mechanisms, as an adaptive response to OS which could not be suppressed by the class I (non-enzymatic) and II (enzymatic) defenses. Failure to suppress OS by those three classes of antioxidant defenses may lead to persistent strong OS and, through suppressing the expression of inhibitory interneurons, may cause prolonged excessive glutamate and/or DA release, leading to excitation toxicity (ET) and additional deterioration of the brain function.

It should be noted that while excessive activation of NMDA-R triggers neuronal degeneration through intracellular OS and resulting MD [67], NMDA-R activation at subtoxic doses has a neuroprotective property due to brain-derived neurotrophic factor (BDNF) release [68-70] and/or blockade of c-Jun N-terminal kinase (JNK) activation [71]. NMDA-R has regulatory redox sites through which the oxidation status regulates the physiological activity of the receptors [72-75]. More recently it has been shown that trans-synaptic stimulation of NMDA-R boosts the class II antioxidant defenses by modifying the thioredoxin-peroxiredoxin system [76]. Thus, it would be conceivable that the brain, the highest energy consuming organ vulnerable to OS, has a third class of antioxidant defenses (‘class III defenses’) in which neurotransmissions are involved, and where trans-synaptic activation of NMDA-R as well as DA-R may occur, through unknown pre-synaptic redox regulation mechanisms, as an adaptive response to OS which could not be suppressed by the class I and II defenses. Failure to suppress OS by those three classes of antioxidant defenses may lead to persistent and strong OS and through suppressing the expression of inhibitory interneurons, may cause prolonged excessive glutamate and/or DA release, leading to excitation toxicity and additional deterioration of the brain function (Figure 1).

#### Enhanced carbonyl stress (CS) in SCZ and the MGM

Recently Arai et al. [77] reported that a subset (47%; N = 21) of patients with SCZ (N = 45) exhibit CS with high plasma pentosidine levels (>mean +2SDs of controls), without underlying diabetes mellitus or chronic kidney disease, the two major causes of elevated AGEs (advanced glycation end-products). In addition, marked decreases in serum pyridoxal levels were found in 50% (N = 11) of those patients with CS. Since pyridoxal is a reactive carbonyl compounds (RCOs) scavenger, decreased pyridoxal levels may be due to the consumption of pyridoxal during prolonged CS. However, the molecular mechanisms underlying the CS in those patients remain unclear.

They also identified 2 rare frameshift variants of loss-of function type in *GLO1* that encodes glyoxalase 1 (GLO1), a detoxifying enzyme of RCOs. While marked CS characterized by elevated AGEs and decreased pyridoxal was associated with those variants in patients with SCZ, it was not in healthy controls. Then, how did these rare variants of *GLO1*cause CS only in patients with SCZ?

A number of enzymatic pathways including glyoxalase pathway contribute to the detoxification of RCOs, and redox coenzymes (reduced GSH and NAD(P)H) are particularly important elements for the activity of these pathways [78]. On the other hand, it has been well known that OS increases RCOs production [78]. Therefore, enhanced OS and secondarily induced alteration of redox coenzyme homeostasis may cause CS in the affected population (Figure 1). And while these missense variants in *GLO1* may cause marked CS in patients with SCZ, they may not cause CS in healthy controls because of normal redox coenzyme activities and lack of enhanced OS.

In this case, the rare frameshift variants of *GLO1* detected by Arai et al. must be FGs. Indeed, patients carrying those rare variants have been severely affected and treatment-resistant cases while the frequency of those variants was not higher in patients with SCZ (5/1761 = 0.28%) than in healthy controls (10/1921 = 0.52%) [77].

### Predictions of the hypothesis

This hypothesis predicts: (1) a higher maternal transmission of SCZ (**H1**), (2) positive associations between PGs and SCZ as well as negative associations between FGs and SCZ (**H1**), and (3) various pathogenic alterations such as genomic instability, aberrations in neuromuscular development, and brain dysfunction, that can be caused by disturbed OXPHOS and enhanced OS in predisposed individuals (**H1 + H2**). In addition, the hypothesis predicts a possible therapeutic effect of ROS scavengers (**H2**).

Most of these predictions seem to be consistent with major epidemiological findings and the results of genetic studies to date.

#### The mode of transmission

The hypothesis (**H1**) predicts *a higher maternal transmission*. Although there has been no convincing evidence for an exclusively maternal transmission of SCZ, several reports suggest a higher maternal transmission of SCZ [79-82].

It also predicts that *a certain proportion of patients are sporadic cases* due to de novo mutation in the mtDNA. Given the average mutation rate in the mtDNA (4.3 × 10^-3^) and the overall prevalence of SCZ (~0.7%), the expected proportion of de novo sporadic cases in the affected population is ~0.6 (= 0.0043/0.007). This figure accords with the results of the epidemiological studies, and the fact that 60% of patients with SCZ have no affected first- and second-degree relatives [1].

Some researchers have hypothesised that SCZ is associated with *de novo* mutations arising in paternal germ cells [83-87]. This is based on the observation (‘paternal age effect’) that the risk of SCZ in the offspring seems to increase as the paternal age advances from 20 years to over 50 years. However, the difference in the mean age of fathers of affected and unaffected individuals is not very large (< 1.7 years) [83,86]. Furthermore, there has always been increased risk of SCZ for the offspring of younger men (< 21 years) [83,86,87] and younger women (< 20 years) [86]. Therefore, major roles of paternally derived mutations in SCZ seem to remain unsubstantiated. Indeed, no available data can exclude the possibility that the ‘paternal age effect’ has a ‘maternal origin’; while women in many countries today may be expected to bear children after the age of 20 years and not to marry much older or younger men unless the men have special socio-economic benefits, a certain proportion of women genetically predisposed to SCZ might behave differently.

It should be noted that in the famous twin study by Gottesman and Bertelsen [88] that included almost equal numbers of male and female monozygotic twins discordant for SCZ, most schizophrenic twins whose offspring were affected were females (12 out of 14), implying that the transmission was mainly via females $\left(P={\;}_{14}C_{12}\times 0.5^{14}+{\;}_{14}\;C_{13}\times 0.5^{14}+{\;}_{14}\;C_{14}\times 0.5^{14}<0.007\right)$.

#### Every SCZ-associated gene is either a PG for SCZ or a PG-linked gene

In the MGM, G-G interactions between the nuclear genome and the pathogenic mitochondrial genome give rise to SCZ-associated PGs that decrease the risk and the severity of SCZ. Thus, the hypothesis (**H1**) predicts that *every SCZ-associated gene is either a PG for SCZ or a PG-linked gene*.

Cys 311 of DRD2 polymorphism and ‘high risk’ variants of *NRG1DTNBP1*, and *NPY* may be such examples because schizophrenics carrying those variants exhibit less negative symptoms [89-91]. Unfortunately, most association studies to date lack sufficient clinical data about the onset and the clinical symptoms of subjects to determine the nature of the SCZ-associated variants detected.

#### An apparent signature of positive selection in SCZ-associated genes

Since the positive selection of the SCZ-associated alleles (= protective alleles) mentioned above occurs only in the predisposed matrilineal pedigrees, a ubiquitous subpopulation in humans, frequencies of those alleles may not be as high as if the selection had occurred *recently* in the general population. Thus the hypothesis (**H1**) predicts that *every SCZ-associated nuclear gene has an apparent signature as if it has been subject to positive selection in the recent evolutionary history of humans*.

Two recently published reports [92,93] seem to concur with this prediction. On the other hand, the NGM predicts that every SCZ-associated nuclear gene has an apparent negative selection signature due to the strong negative selection pressure, which is contrary to these reports.

#### Sex differences and the protective effect of estrogen in SCZ

The hypothesis (**H2**) predicts that *endogenous antioxidants exhibit a protective effect against SCZ*, and may give a plausible explanation for sex differences in the disease.

A consistent and specific finding for SCZ is that the age at onset is significantly lower in males than in females [1,94]. SCZ starts earlier on average in males and reaches its peak between 15 and 25 years of age; whereas, in females it occurs almost between 20 and 30 years of age and shows a less steep curve after that age. It also appears that women are vulnerable to relapses or first episodes of SCZ in the perimenoposal period (the second peak of onset for females) [94], when estrogen production diminishes. A close association between premenstrual or menstruation phase and exacerbation of the illness in females has been well documented [94]. In addition, less negative symptoms, less brain morphological changes, and a better response to neuroleptics are relatively consistent findings in female patients with SCZ [95].

From these observations, one may infer that estrogen protects predisposed females [94]. However, the exact mechanism for this finding remains unclear [94,95]. The hypothesis (**H2**) may provide a plausible explanation, because estrogen has been shown to exhibit antioxidant activity due to its intrinsic antioxidant structure that lies in the phenolic moiety of the steroidal compound [96], to increase antioxidant enzyme activities [97,98], and to have a neuroprotective effect against OS [96,99]. Furthermore, mitochondrion has estrogen binding sites [100,101] and estrogen increases mitochondrial efficiency and reduces intracellular OS [102].

#### Prenatal risk factors for SCZ

The hypothesis (**H2**) predicts that *early-life exposure to environments that induce OS can increase the risk of later development of SCZ in the predisposed population*.

Indeed, prenatal environmental factors such as severe nutritional deficiency [103], exposure to increased homocysteine [104] or lead [105], and infection of influenza virus [105-107] and Toxoplasma gondii [108] have been suggested to increase the risk for SCZ. More recently, it has been suggested that central nervous system infections of cytomegalovirus or mumps virus in childhood may also increase the risk for SCZ [109]. All of these factors have been shown to affect mitochondria, inducing strong intracellular OS and/or apoptosis [110-122].

#### Increased obstetric complications in the birth of patients with SCZ

It has been suggested that MD may be involved in the etiology of preeclampsia [123-125]. Furthermore, a high incidence of preeclampsia, eclampsia, and stillborn infants has been observed in a family with a known mitochondrial disorder [126]. Folgero et al. [127] demonstrated two separate mtDNA point mutations in two families having a high incidence of preeclampsia and eclampsia.

Therefore, the hypothesis (**H1**) predicts that *the risk of preeclampsia and stillbirth may be increased in the birth of patients with SCZ as well as in the pregnancies of women with SCZ*.

There has been a body of evidence used to account for an increased risk of obstetric complications (OCs) in the birth of patients with SCZ [128,129]. A meta-analysis of population-based data [129] found significant estimates for three main categories of OCs: (1) complications of pregnancies, (2) abnormal fetal growth and development, and (3) complications in delivery. Among all, preeclampsia was the strongest individual risk factor detected in the largest single population-based cohort study to date [128]. Although obstetrical events in SCZ are often considered as having a direct causative effect, no available data have been able to refute the theory that these events are merely markers of some other causal process [130], such as MD implicated in this hypothesis **(H1**). An excess of stillbirths and neonatal deaths among women with SCZ has been reported by several investigators [131-133].

#### Low comorbidity between SCZ and rheumatoid arthritis (RA)

The hypothesis (**H1**) predicts that *diseases predisposed by FGs, if present, would be negatively associated with SCZ*. RA could be one such candidate.

OS plays a role in the pathogenesis of RA (for review, see [134]). In addition, it has been shown that chronic OS in the synovial T lymphocytes is not secondary to exposure to environmental ROS, but instead originates from intracellularly produced ROS [135]. Therefore, a presumed susceptibility gene for RA that may cause intracellular OS in several cell lines could be a FG for SCZ in the MGM, and is likely negatively associated with SCZ.

Indeed, robust evidence shows a negative association between SCZ and RA; although, the exact mechanism is unknown [1,136-138]. According to the NGM, several hypotheses have been proposed, including that pathogenic genes for SCZ may be protective genes for RA and vice versa [1,136-138].

Thus the MGM may offer a new explanation for the low comorbidity and provide an additional prediction: *most of patients with both of SCZ and RA would be females because the survival rate of males in early life stages must be remarkably reduced due to lack of protection by estrogen, and show more negative symptoms, poorer response to neuroleptic medication, and/or more pronounced morphological changes in the brain*.

#### Genomic or epigenomic instability

It has been shown that endogenous mitochondrial OS can induce many types of DNA damage including double strand breaks, end-to-end fusions, base and sugar modifications, DNA-protein cross-links, and gross chromosomal rearrangements [139,140]. Therefore, the hypothesis (**H1**) predicts that *the enhanced OS may cause genomic or epigenomic instability during meiosis and/or early phase of ontogeny, producing increased rates of random point mutations and/or structural variants of the nuclear genome in the affected population*. In addition, *genomic instability may be more pronounced in male patients due to lack of antioxidant protection by estrogen*.

There have been numerous reports of associations between SCZ and chromosomal abnormalities including fragile sites, reciprocal translocations, inversions, insertions, deletions, disomy and trisomy in many autosomes, and sex chromosome aneuploidies [141]. However, with the exception of 22q11 deletion, none of these have been consistently replicated.

A popular explanation in the NGM may be that most of these structural variants are coincidental findings of no clinical significance. Alternatively, those alterations may indicate genomic instability in SCZ. An increased risk of SCZ in individuals with 22q11 deletion [142,143] might be due to haplodeficiency of presumptive PGs of gain-of-function type and/or presumptive FGs of loss-of-function type aggregated on 22q11.

More recently, it has been reported that rare structural variants such as microdeletions or microduplications of sizes ranging from 100 kb to 15 MB throughout the genome are more frequent among individuals with SCZ than unaffected individuals [144]. While many of those structural variants duplicate or delete genes in neurodevelopmental pathways, one third of those do not disrupt genes, leaving their role in causation of the disease unwarranted. Another recent report [145] has shown that *de novo* copy number mutations are increased in sporadic SCZ. However, the cytobands of those copy number mutations are diverse among the affected individuals and their roles in the pathogenesis still remain unclear. Therefore, no available data can refute the possibility that those structural variants and copy number mutations are not the cause of SCZ but the results of the genomic instability in SCZ predicted by our hypothesis.

Indeed, direct measure of the *de novo* mutation rates shows an increased mutation rate in SCZ [146], and genomic or epigenomic instability has been suggested in SCZ [147]. Furthermore, it has been shown that blood cells from patients with SCZ present a higher rate of folate-sensitive fragile sites, and that male patients exhibit twice as many fragile sites as females while there are no age effects [148]. This sex difference may indicate that increased fragile sites expression (genomic instability) is the results of enhanced OS associated with SCZ.

#### Inconsistency in the results of the association studies on the nuclear genome

Results of association studies based on the NGM have been inconsistent, and SCZ-associated genes including copy number variations differ across populations or even across individuals of the same ethnicity [19,20,144,145].

The only plausible explanation for this inconsistency in the NGM is that there is an extreme heterogeneity in the genetic basis of SCZ.

On the other hand, these perplexing results of the association studies to date accord with a specific prediction of the hypothesis: in the MGM, a disease-associated allele is likely to be a PG, and may show a stronger association with milder cases and unaffected siblings but not with severe cases, while a negatively associated allele is likely to be a FG, and may show a positive association with severer early-onset cases. Therefore, *an association detected in a study with mildly affected subjects may not be supported in studies with severely affected subjects, and an association detected in a study with severely affected subjects may not be supported in studies with mildly affected subjects*. This is a specific prediction of the hypothesis (**H1**).

#### Therapeutic effects of ROS scavengers

In this hypothesis (**H1 + H2**), OS and secondarily induced CS cause pathogenic alterations in SCZ. Therefore, it predicts that *ROS scavengers have therapeutic effects on SCZ*.

Indeed, adjuvant therapy using neuroleptics and antioxidants such as omega-3 poly unsaturated fatty acids, ascorbic acid, α-tocopherol or N-acetyl cysteine have been shown to improve clinical outcomes of patients with SCZ (for review see [18]). However, the most promising therapeutic antioxidant for SCZ may be molecular hydrogen, which rapidly diffuses across membranes and selectively reacts with cytotoxic ROS, but not with other ROS that play physiological roles [149]. This prediction can be easily tested in the near future.

### Possibilities and limitations of the hypothesis

This new hypothesis seems to explain various and specific aspects of SCZ and somewhat perplexing results of association studies to date. It meets the four criteria for evolutionary hypotheses of SCZ proposed by Brüne [4] because it: (1) provides a convincing mechanism for the preservation of genes in the human gene pool associated with SCZ, (2) explains potential sex differences, (3) accounts for the multifaceted symptomatology, and (4) is consistent with neuropsychological, developmental and evolutionary findings regarding the human brain.

However, there are several limitations to this hypothesis. First of all, it is only a theory that lacks direct and compelling evidence. Especially, the assumption of the class III antioxidant defenses in the brain should be tested in neurophysiological studies in the future. Second, the exact mechanism of discordance in monozygotic twins remains unclear. Although the hypothesis may explain the discordance due to different mutation loads in the first ovum cleavage, it still requires future investigation. Third, while there have been several studies that report new variants in the mitochondrial genome associated with SCZ [150-156], there have been no consistent findings on the pathogenic variants in the mitochondrial genome. However, it should be noted that any missense variant in the mitochondrial genome can be a pathogenic genome in the MGM because it may contribute to cause disturbed OXPHOS and enhanced OS. There could be heterogeneity in the genetic basis of SCZ in the MGM, as are the cases with known mitochondrial diseases [157].

Although a recent study failed to detect any pathogenic mitochondrial genome in the blood cells [158], it cannot necessarily deny the possibility of the MGM because of the heteroplasmy of the mitochondrial genome. It has been well known that pathogenic mitochondrial genome cannot necessarily be detected in the blood cells in known mitochondrial diseases [159]. Therefore, other cell lines such as muscles, neurons, or hair follicles should be examined to detect pathogenic variants in the mitochondrial genome in the future.

## Conclusions

Genetic research of SCZ based on the NGM has been one of the most active areas in psychiatry for the past two decades. Although this effort is ongoing, results of association studies based on the NGM have been disappointing, or rather perplexing. No particular susceptibility gene that accounts for a large proportion of heritability has been identified by association studies. The results of association studies have been inconsistent, and SCZ-associated variants including copy number variations differ across populations or even across individuals of the same ethnicity.

The central paradox of SCZ genetics and the results of association studies to date argue against the NGM for SCZ today, and in its place the MGM is emerging as a viable option to account for genomic and pathophysiological research findings involving this enigmatic disorder.

## Appendix A. Deduction of the persistence criterion

Deduction of the P-criterion is explained in detail elsewhere[11]. Therefore, we present here the essence of the method. At first we describe our three basic assumptions.

## An ideal human population

We assume here a random-mating human population with a sufficiently large effective population size at equilibrium, where negative selection pressures on the susceptibility alleles for SCZ are predominant and the effect of genetic drift is negligibly small. The prevalence *p* of SCZ in this ideal human population is assumed to be stable across generations by mutation-selection balance. Therefore, the gene frequency in the general population (*m*_*G*_) is given in terms of the gene frequencies in the affected population (*m*_*A*_) and in the unaffected population (*m*_*U*_):

(1) $$m_{G}=pm_{A}+(1-p)m_{U}\text{,}\;orm_{A}-m_{G}=(1-p)d.(d\equiv m_{A}-m_{U})$$

## Mutation-selection balance in each risk locus

We assume here that the total number of population frequencies of the pathogenic alleles at *each risk locus* is preserved by mutation-selection balance. Therefore, $-\Delta m_{G}$, the cross-generational reduction in the frequency of a pathogenic allele should not be more than the rate of mutations that produce pathogenic variants at the locus. On the other hand, since mutations at the locus include mutations of two directions that produce pathogenic or non-pathogenic variants, the mutation rate at the locus should be greater than the rate of mutations that produce pathogenic variants at the locus. Thus we have:

(2) $$\mu >-\Delta m_{G}\text{.}$$

## Multifactorial threshold model

We assume the multifactorial threshold model, in which quantitative traits such as liability to the disease are determined by multiple genetic and non-genetic factors including a stochastic and/or an epigenetic effect. Under this assumption, the relative fitness as a quantitative trait in the affected population is determined by multiple factors and approximately follows a gamma distribution with a mean(1–*s*). (*s* is the selection coefficient of SCZ; the mean relative fitness in the normal population is defined as unity.)The distribution curve of the fitness in the affected subpopulation with a SCZ-associated allele M never shifts to the right unless M has a strong protective effect (i.e. the effect of elevating reproductive fitness by reducing severity of and liability to the disease). Therefore, we can assume that *s*_*M*_, the selection coefficient in the affected subpopulation with a SCZ-associated allele M, is not smaller than *s*$\left(s\leq s_{M}<1\right)$ for a susceptibility allele. The inequality *s*>*s*_*M*_ implies that M is a protective gene that reduces the severity and the risk of the disease.No special assumptions are required on the allelic structure in each locus, or penetrance of each susceptibility gene, and possible interactions among the loci.Now we proceed to deduce the persistence criteria. From the assumptions, $m\prime_{G}$, the population frequency of the SCZ-associated allele M in the next generation, is given by:$m\prime_{G}=\frac{p\cdot m_{A}\cdot (1-s_{A})+(1-p)\cdot m_{U}\cdot 1}{p\cdot (1-s_{M})+(1-p)\cdot 1}=\frac{m_{G}-s_{M}\;pm_{A}}{1-s_{M}p}$. Therefore, from (A1), the reduction of the population frequency of the SCZ-associated allele M per generation is

(3) $$-\Delta m_{G}=m_{G}-m\prime_{G}=\frac{s_{M}\;p(m_{A}-m_{G})}{1-s_{M}p}=p(1-p)d\cdot \frac{s_{M}}{1-s_{M}p}\text{.}$$

From (A2) and (A3) we have

(4) $$\mu >p(1-p)d\cdot \frac{s_{M}}{1-s_{M}p}\text{.}$$

Since $\frac{s_{M}}{1-s_{M}\;p}$ is monotonically increasing for *s*_*M*_$\left(0<s_{M}<1\right)$ and $s\leq s_{M}<1$ holds for susceptibility allele M, we have

(5) $$\mu >p(1-p)d\cdot \frac{s}{1-sp},or\frac{(1-sp)\mu }{(1-p)sp}>d\text{.}$$

Thus we have the criterion for a susceptibility gene:

(6) d<ν,

where *ν* is defined as $\nu \equiv \frac{(1-sp)\mu }{(1-p)sp}$.From the observation (A5), we can see that *d*≥*v* implies *s*>*s*_*M*_ for any SCZ-associated variant M which is sustained by mutation-selection balance.

## Appendix B. Mutation rates and parameter-estimations for SCZ

Mutation rates on autosomes and the X chromosome almost always fall within the range between 10^-6^ and 10^-4^ per locus per generation (usually < 10^-5^; one generation = 20 years) [13,14] and can be approximated by a linear function of the parental age at least under 30 years for maternal age and under 40 years for paternal age [160]. Large-sampled cohort studies in Israel, Sweden and Denmark show that the mean age of parents in the general population is ~ 28 years for mothers and ~31 years for fathers; the mean age of both parents is < 29.6 years [83,86]. Therefore we can assume: $10^{-6}<\mu <\frac{29.6}{20}\times 10^{-4}=1.48\times 10^{-4}$. According to the epidemiological data by Haukka et al. [8], the estimated values for *p* and *s* are$p=1.29\times 10^{-2}$and $s=6.54\times 10^{-1}$. Therefore, we have $\nu =1.76\times 10^{-3}$for the average mutation rate $\left(1.48\times 10^{-5}\right)$$\nu =1.76\times 10^{-2}$for the highest mutation rate $\left(1.48\times 10^{-4}\right)$, and $\nu =1.76\times 10^{-4}$for a relatively low mutation rate $\left(1.48\times 10^{-6}\right)$.

## Appendix C. Estimation of the required sample size of a GWAS

Concerning the required sample size 2 *N* (*N* case–control pairs) and the power 1–*β* of an association study, we have the well-established formula [161]:$N≅\frac{1}{2}\;{\left(\frac{z{*}_{\alpha }\sqrt{2\times (1-x)}+z_{\beta }\gamma }{d}\right)}^{2}$, where *x* (population frequency of the allele), *d* (case–control difference of allele frequencies) and *γ*^2^ are defined as: $x\equiv \frac{1}{2}(m_{A}+m_{U})$, $d\equiv m_{A}-m_{U}$, and $\gamma^{2}\equiv m_{A}(1-m_{A})+m_{U}(1-m_{U})=2x(1-x)-\frac{1}{2}d^{2}$.For the average mutation rate$\mu =1.48\times 10^{-5}$, we have $\nu =1.76\times 10^{-3}$. Supposing0.0005<x<0.9995, we have $2x(1-x)>0.9995\times 10^{-3}$. From the persistence criterion, we have:$\frac{1}{2}d^{2}<\frac{1}{2}\nu^{2}<1.6\times 10^{-6}<2x(1-x)\times 0.002$. Therefore, we have the following approximation with an error smaller than 0.2%:

(7) $$\gamma^{2}=2x(1-x)-\frac{1}{2}d^{2}≅2x(1-x),\text{ or}\;\gamma ≅\sqrt{2x(1-x)}\text{.}$$

Thus, we have$N≅\frac{1}{2}{\left(\frac{z{*}_{a}\sqrt{2x\left(1-x\right)}+z_{\beta }\gamma }{d}\right)}^{2}≅{\left(\frac{z{*}_{a}+z_{\beta }}{d}\right)}^{2}\;x\left(1-x\right)>{\left(\frac{z{*}_{a}+z_{\beta }}{v}\right)}^{2}x\left(1-x\right)$.

Let us calculate the required sample size in a GWAS $\left(\alpha =2.5\times 10^{-7},1-\beta =0.80\right)$. Since we have $z{*}_{0.00000025}+z_{0.2}=5.99$,$N>{\left(\frac{z_{\alpha }^{*}+z_{\beta }}{\nu }\right)}^{2}x(1-x)={\left(\frac{5.99}{1.76\times 10^{-3}}\right)}^{2}x(1-x)=2.90\times 10^{6}$ for *x=*0.5. Therefore, more than 2.9 million case–control pairs are required in a genome-wide association study with a power of 0.8 to detect a susceptibility variant of the average mutation rate and a population frequency between 0.0005 and 0.9995.Similarly we can see that more than 29,000 case–control pairs are required in a genome-wide association study with a power of 0.8 to detect a susceptibility variant of the highest mutation rate $\left(\mu =1.48\times 10^{-4}\right)$ and a population frequency between 0.005 and 0.995.Finally, let us consider the case with a relatively low mutation rate $\mu =1.48\times 10^{-6}$, which corresponds to $\nu =1.76\times 10^{-4}$. In this case, more than 290 million case–control pairs are required in a GWAS with a power of 0.8 to detect a susceptibility variant of a population frequency between 0.000005 and 0.999995.

## Abbreviations

AGEs: Advanced glycation endproducts; CS: Carbonyl stress; DA: Dopamine; DA-R: Dopamine receptor; DRD2: Dopamine D2 receptor; ET: Excitation toxicity; FG: Facilitating gene that increases the risk and the severity of schizophrenia in the presence of a pathogenic mitochondrial genome; G-E interaction: Gene-environment interaction; G-G interaction: Gene-gene interaction; Glu: Glutamate; GSH: Glutathione; GWAS: Genome-wide association study; MD: Mitochondrial dysfunction; MGM: Mitochondrial genome model; mtDNA: Mitochondrial DNA; NADH: Reduced nicotinamide adenine dinucleotide; NADPH: Reduced nicotinamide adenine dinucleotide phosphate; ncDNA: Nuclear DNA; NGM: Nuclear genome model; NMDA-R: N-methyl-d-aspartate-receptor; OR: Odds ratio; OS: Oxidative stress; OXPHOS: Oxidative phosphorylation; PG: Protective gene that decreases the risk and the severity of schizophrenia; ROS: Reactive oxygen species; RCO: Reactive carbonyl compound; rRNA: Ribosomal RNA; tRNA: Transfer RNA; SCZ: Schizophrenia; Z*α: The two sided α point of the standard normal curve; Zβ: The upper β point of the standard normal curve.

## Competing interest

We declare no conflict of interest.

## Authors’ contribution

ND and YH conceived the study and wrote the first draft. YH, MI, TY, TI, MA, CU and HT revised the manuscript through discussion with ND. All authors read and approve the final manuscript.
